# Role of Cystathionine β-Synthase and 3-Mercaptopyruvate Sulfurtransferase in the Regulation of Proliferation, Migration, and Bioenergetics of Murine Breast Cancer Cells

**DOI:** 10.3390/antiox12030647

**Published:** 2023-03-05

**Authors:** Sidneia Sousa Santos, Larissa de Oliveira Cavalcanti Peres Rodrigues, Vanessa Martins, Maria Petrosino, Karim Zuhra, Kelly Ascenção, Abhishek Anand, Reham Mahmoud Abdel-Kader, Mohamed Z. Gad, Carole Bourquin, Csaba Szabo

**Affiliations:** 1 Department of Medicine, Division of Infectious Diseases, Escola Paulista de Medicina, Federal University of São Paulo (EPM/UNIFESP), São Paulo 04023, Brazil; 2 Chair of Pharmacology, Section of Science and Medicine, University of Fribourg, 1700 Fribourg, Switzerland; 3 Pharmacology and Toxicology Department, Faculty of Pharmacy and Biotechnology, German University in Cairo, Cairo 11511, Egypt; 4 Department of Biochemistry, Faculty of Pharmacy and Biotechnology, German University in Cairo, Cairo 11511, Egypt; 5 School of Pharmaceutical Sciences, Institute of Pharmaceutical Sciences of Western Switzerland, Department of Anaesthesiology, Pharmacology, Intensive Care and Emergency Medicine, University of Geneva, 1211 Geneva, Switzerland

**Keywords:** hydrogen sulfide, bioenergetics, gasotransmitters, nitric oxide, proliferation, migration, mitochondria

## Abstract

Cystathionine β-synthase (CBS), CSE (cystathionine γ-lyase) and 3-mercaptopyruvate sulfurtransferase (3-MST) have emerged as three significant sources of hydrogen sulfide (H_2_S) in various forms of mammalian cancer. Here, we investigated the functional role of CBS’ and 3-MST’s catalytic activity in the murine breast cancer cell line EO771. The CBS/CSE inhibitor aminooxyacetic acid (AOAA) and the 3-MST inhibitor 2-[(4-hydroxy-6-methylpyrimidin-2-yl)sulfanyl]-1-(naphthalen-1-yl)ethan-1-one (HMPSNE) were used to assess the role of endogenous H_2_S in the modulation of breast cancer cell proliferation, migration, bioenergetics and viability in vitro. Methods included measurements of cell viability (MTT and LDH assays), cell proliferation and in vitro wound healing (IncuCyte) and cellular bioenergetics (Seahorse extracellular flux analysis). CBS and 3-MST, as well as expression were detected by Western blotting; H_2_S production was measured by the fluorescent dye AzMC. The results show that EO771 cells express CBS, CSE and 3-MST protein, as well as several enzymes involved in H_2_S degradation (SQR, TST, and ETHE1). Pharmacological inhibition of CBS or 3-MST inhibited H_2_S production, suppressed cellular bioenergetics and attenuated cell proliferation. Cell migration was only inhibited by the 3-MST inhibitor, but not the CBS/CSE inhibitor. Inhibition of CBS/CSE of 3-MST did not significantly affect basal cell viability; inhibition of 3-MST (but not of CBS/CSE) slightly enhanced the cytotoxic effects of oxidative stress (hydrogen peroxide challenge). From these findings, we conclude that endogenous H_2_S, generated by 3-MST and to a lower degree by CBS/CSE, significantly contributes to the maintenance of bioenergetics, proliferation and migration in murine breast cancer cells and may also exert a minor role as a cytoprotectant.

## 1. Introduction

Hydrogen sulfide (H_2_S) is an endogenous gaseous transmitter which has been implicated in multiple regulatory processes in mammals [1,2]. There are three principal enzymatic sources of H_2_S in various cancer cells: cystathionine β-synthase (CBS), cystathionine γ-lyase (CSE) and 3-mercaptopyruvate sulfurtransferase (3-MST) [1,2].

Over 10 years, a novel concept emerged in cancer biology, demonstrating that various cancer cells upregulate endogenous H_2_S-producing enzymes and utilize H_2_S to support various cancer cell functions, such as cell proliferation, cytoprotective signaling, cellular bioenergetics, and angiogenesis [3,4].

In the current study, we have characterized the expression of H_2_S-producing and H_2_S-metabolizing enzymes in the murine breast cancer cell line EO771. In addition, using pharmacological approaches, we have assessed the role of CBS and 3-MST catalytic activity in the maintenance of various fundamental cellular functions in vitro including cellular bioenergetics, cell proliferation, migration and cell viability. To this aim, we have utilized the standard CBS/CSE inhibitor aminooxyacetic acid (AOAA) [5] and the recently discovered, selective 3-MST inhibitor, 2-[(4-hydroxy-6-methylpyrimidin-2-yl)sulfanyl]-1-(naphthalen-1-yl)ethan-1-one (HMPSNE) [6].

The data presented indicate that in EO771 cells, the 3-MST/H_2_S system, and—to a lower extent—the CBS/H_2_S system contribute to the maintenance of cellular bioenergetics, cell proliferation and cell migration, and that the 3-MST system may also serve a minor cytoprotective role against oxidative stress.

## 2. Materials and Methods

### 2.1. Cell Culture

The EO771 murine epithelial-like carcinoma cell line (ATCC #CRL-3461; American Type Culture Collection, Manassas, VA, USA) was grown in DMEM culture medium containing 4.5 g/L D-glucose, supplemented with 10% fetal bovine serum (FBS, Hyclone, Pittsburgh, PA, USA), 100 units/mL of penicillin and 100 µg/mL of streptomycin and 2% of HEPES (GE Healthcare, Pittsburgh, PA, USA). For experiments and sub-culturing, cells were rinsed with PBS and detached from T75 flasks by incubating with 0.25% (*w*/*v*) trypsin 0.53 mM EDTA for 2 min at 37 °C followed by resuspension in culture medium.

### 2.2. Western Blotting

The EO771 cell suspension was centrifuged for 5 min at 400× *g* and the pellet was resuspended in RIPA Lysis and Extraction Buffer (Thermo Scientific, Waltham, MA, USA) complemented with Halt™ Protease and Phosphatase Inhibitor Cocktail (Thermo Scientific) just prior use. The protein concentration was determined with Bradford assay (employing Pierce™ Coomassie Plus Assay Reagent—Thermo Scientific) and an Infinite 200 Pro reader (Tecan, Männedof, Switzerland). Samples were prepared for gel electrophoresis in Bolt™ LDS Sample Buffer (4X) (Invitrogen) and Bolt™ Reducing Agent (10X) (Invitrogen, Thermo Scientific) according to manufacturer’s instructions, loaded in Bolt™ 4–12% Bis-Tris Plus Gels (Invitrogen, Thermo Scientific) and ran at 120 V.

Proteins were transferred onto a PVDF (polyvinylidene difluoride) membrane by dry transfer using the iBlot™ 2 Device and Transfer Stacks (Invitrogen). The membrane was blocked with 5% Milk in TBS/0.1% Tween (TBST/5% Milk). Protein expression was evaluated by Western blotting using anti-CBS (14782S) 1:250 from Cell Signaling (Beverly, MA, USA), anti-3-MST 1:500 (ab154514), anti-CSE 1:1000 (ab151769), anti-TST 1:1000 (ab231248) from Abcam (Cambridge, UK), anti-ETHE-1 1:1000 (GTX109095) from GeneTex, anti-SQR antibody 1:1000 (HPA017079) and anti-beta-actin antibody (1:3000) was obtained from Sigma-Aldrich Chemie Gmbh (Munich, Germany). Incubations were conducted overnight at 4 °C under agitation. The membranes were subsequently washed with TBST, and incubated for 1 h at room temperature (RT) with the secondary antibodies anti-rabbit IgG or anti-mouse IgG, HRP-linked antibody (Cell Signaling, Beverly, MA, USA) diluted 1:5000 in TBST/5% Milk. Amersham ECL™ Prime Western Blotting Detection Reagent (GE Healthcare, Pittsburgh, PA, USA) was used for detection; chemiluminescence was measured and quantified with the Azure Imaging System 300 (Azure Biosystems, Dublin, CA, USA).

### 2.3. Viability and Metabolic Assay

EO771 cells were seeded in sterile 96-well plates (20,000 cells/well) and incubated at 37 °C and 5% CO_2_. Cells were treated with aminooxyacetate hemihydrochloride (AOAA, Sigma-Aldrich, St. Louis, MO, USA) (300 µM) or HMPSNE (MolPort, Riga, Latvia) (200 µM) for 2 h or 24 h followed by 2 h with increasing concentrations of H_2_O_2_. Then, 50 µL of the supernatant from each well was then transferred to another plate. The activity of lactate dehydrogenase (LDH) in the supernatant was used to estimate the degree of cell necrosis [7].

Cells were then subjected to the MTT assay, a method to assess cell viability/mitochondrial activity based on the activity of the cell’s NADH-dependent cellular oxidoreductase enzyme activity [7]. Cells were placed in 50 µL/well of serum-free medium supplemented with MTT reagent (Abcam, Cambridge, UK) and were incubated for 3 h at 37 °C and 5% CO_2_. Formazan produced by cells with active metabolism was solubilized in 150 µL/well of MTT solvent by mixing well and shaking in an orbital way for 60 s at RT protected from light. Absorbance was measured at 590 nm using a Tecan Infinite 200 Pro reader. The LDH assay was performed as described using the Pierce LDH Cytotoxicity Detection Kit Plus (Roche, Mannheim, Germany). Briefly, the LDH reaction mixture was prepared according to the manufacturer’s instructions, and 50 µL/well was added to the supernatants. The plate was incubated for 30 min at room temperature. The reaction was stopped with 50 µL/well of Stop Solution. The plate was shaken in an orbital way for 60 s by Infinite 200 Pro reader (Tecan). Finally, absorbance was measured at 490 nm, with absorbance at 680 nm used as background.

### 2.4. Detection of H_2_S Production in Live Cells

EO771 cells were seeded in sterile black 96-well plate with optical bottom at 15,000 cells/well in 100 µL of complete culture medium and incubated overnight at 37 °C and 5% CO_2_. The day after cells were treated with different concentrations of AOAA or HMPSNE as described above. After 24 h treatment, H_2_S generation in live cells was measured using the 7-azido-4-methylcoumarin (AzMC) assay as described [8]. Briefly, culture medium was replaced with HBSS buffer supplemented with 100 μM AzMC fluorescent dye and further incubated for 1 h. The specific fluorescence of the dye was visualized using a Leica DFC360 FX microscope. Images were captured with Leica Application Suite X software (Leica Biosystems Nussloch GmbH, Germany) and subsequently analyzed with ImageJ software (v. 1.8.0; NIH, Bethesda, MD, USA) and data and graphed with GraphPad Prism 8 (GraphPad Software Inc.; San Diego, CA, USA).

### 2.5. Determination of Cellular Bioenergetics

The Seahorse XFe24 flux analyzer (Agilent Technologies, Santa Clara, CA, USA) was used to estimate cellular bioenergetics of EO771 cells as described [7]. Cells (20,000/well) were seeded on cell culture microplates, incubated for 24 h, followed by treatment with AOAA (300 µM), HMPSNE (200 µM) or its vehicle for 2 h. For analysis of mitochondrial respiration, cells were washed twice with DMEM (pH 7.4) supplemented with L-glutamine (2 mM, Gibco, Thermo Scientific, Waltham, MA, USA), sodium pyruvate (1 mM, Sigma-Aldrich) and glucose (10 mM, Sigma-Aldrich). After 1 h incubation at 37 °C in CO_2_-free incubator, the O_2_ consumption rate (OCR) after oligomycin (1 µM) was used to estimate the ATP production rate. Moreover, carbonyl cyanide-4-trifluoromethoxy phenylhydrazone (FCCP, 0.5 µM) was employed to estimate the maximal mitochondrial respiratory capacity. Electron flux through complex III and I was blocked, respectively, with antimycin A (0.5 µM) and rotenone (0.5 µM). Residual activity in the presence of these inhibitors was considered non-mitochondrial OCR.

For the analysis of glycolytic parameters, cells were treated with AOAA or HMPSNE as above, washed twice with phenol red-free DMEM (pH 7.4) containing L-glutamine (2 mM), sodium pyruvate (1 mM), glucose (10 mM) and HEPES (5 mM). After a 1 h incubation at 37 °C in CO_2_-free incubator, proton efflux rate (PER) from basal and compensatory glycolysis was measured. Rotenone (0.5 µM) and antimycin A (0.5 µM) were used to estimate mitochondrial acidification. At the end of the experimental run, 2-deoxy-D-glucose (50 mM) was used to stop glycolytic acidification.

For the analysis of the glutamine oxidation pathway, after treatment with AOAA and HMPSNE cells were washed twice with DMEM (pH 7.4) supplemented with L-glutamine (2 mM, Gibco), sodium pyruvate (1 mM, Sigma-Aldrich) and glucose (10 mM, Sigma-Aldrich). After 1 h incubation at 37 °C in CO_2_-free incubator, OCR after injection of 0.3 µM bis-2-(5-phenylacetamido-1,2,4-thiadiazol-2-yl)ethyl sulfide (BPTES), an inhibitor of glutaminase, was used to estimate the dependency of cells to use the glutamine oxidation pathway to fuel bioenergetics. Addition of oligomycin (1 µM) and FCCP 0.5 µM, as above, was used to estimate the rate of ATP production and the maximal mitochondrial respiratory capacity, respectively. Eventually, antimycin A (0.5 µM) and rotenone (0.5 µM) were employed to estimate the non-mitochondrial OCR. All data were normalized with total protein content, using the BCA protein assay (Thermo Scientific).

### 2.6. Growth Monitoring, Viability and Metabolic Assay

EO771 cells were seeded in sterile 96-well plate (5000 cells/well) in 100 µL of complete culture medium and incubated over-night at 37 °C and 5% CO_2_. The day after, different concentrations of AOAA or HMPSNE were added as indicated and cell proliferation was monitored for 72 h using the IncuCyte Live Cell Analysis device (20× objective) (Essen Bioscience, Hertfordshire, UK) as described [7]. Cell confluence was recorded every hour by phase-contrast scanning for 72 h at 37 °C and 5% CO_2_ and calculated from the microscopy images.

### 2.7. Migration Assay

EO771 cells were seeded in sterile transparent 96-well plate at 50,000 cells/well in 100 µL of complete culture medium and incubated over-night at 37 °C and 5% CO_2_. The day after, a scratch wound was made in the confluent cell monolayer of each well using the WoundMaker from Essen Bioscience as described [7]. The culture medium was then carefully replaced with indicated AOAA or HMPSNE serial dilutions and the plates were readily placed in IncuCyte device (20× objective) and incubated at 37 °C and 5% CO_2_. Images were acquired every 2 h for up to 48 h to monitor the closure of the wound. Images were analyzed using the IncuCyte ZOOM software to calculate cell confluence over the time.

### 2.8. Statistical Analysis

Data are presented as representative blots or the mean values ± standard error of the 181 mean (SEM) of experiments performed on at least N = 3 experimental days. ANOVA followed by Bonferroni’s multiple comparisons test and One-way ANOVA and Dunnett’s multiple comparisons test were used to analyze the numerical data. A *p* < 0.05 was considered statistically significant. Significance is designated by asterisks signs: * or # for *p* < 0.05, ** for *p* < 0.01 and *** for *p* < 0.001.

## 3. Results

### 3.1. Expression Analysis of H_2_S-Generating and -Metabolizing Enzymes in EO771 Cells

**Figure 1:**
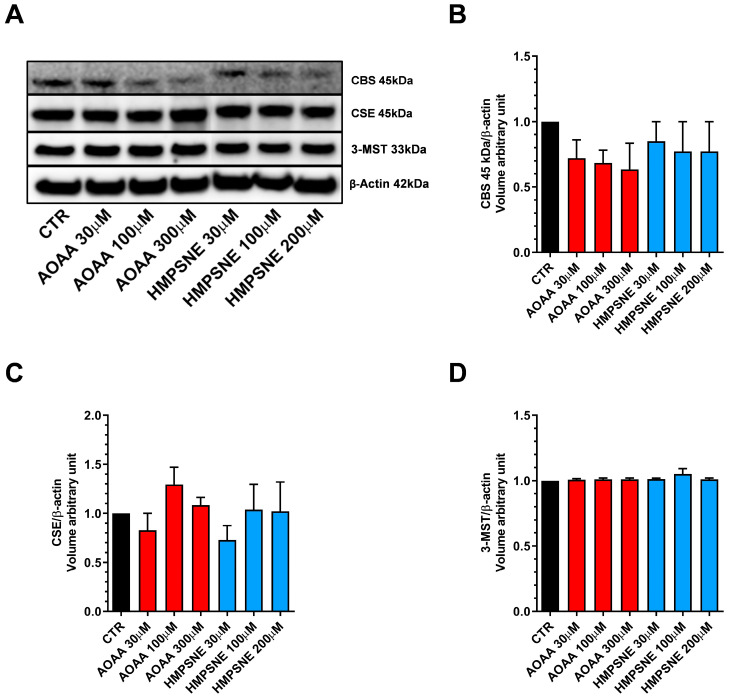
Expression profile of the H_2_S-synthesizing enzymes (CBS, CSE, and 3-MST) in EO771 cells. Expression levels were analyzed by Western blotting. Representative Western blot images of EO771 treated with different concentrations of AOAA or HMPSNE for 24 h and corresponding densitometry analysis (**A**–**D**). β-Actin was used as loading control. N = 4 independent experiments ± SEM.

We have used EO771 cells, which is a murine luminal B mammary cancer cell line (estrogen receptor α negative, estrogen receptor β positive, progesterone receptor positive and ErbB2 positive), originally isolated from a spontaneous tumor in a C57BL/6 mouse [9]. The results demonstrate that the EO771 cells express all three principal H_2_S-generating enzymes, CBS, CSE and 3-MST. For CBS, in these cells, the cleaved form of the enzyme—which does not have the regulatory domain and is constitutively active [10,11]—is the predominant form present in these cells, with full-length CBS not detectable by Western blotting (Figure 1).

**Figure 2:**
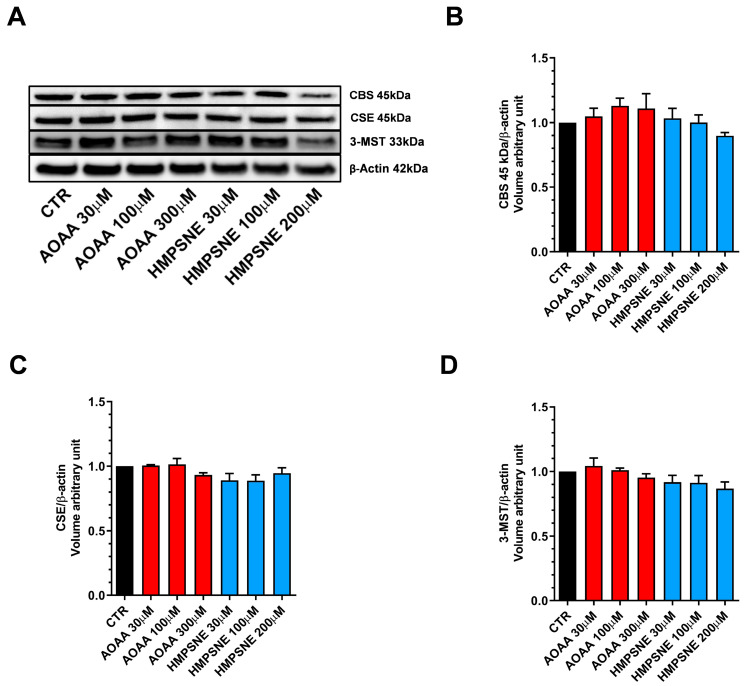
Expression profile of the H_2_S-synthesizing enzymes (CBS, CSE, and 3-MST) in EO771 cells. Expression levels were analyzed by Western blotting. Representative Western blot images of EO771 treated with different concentrations of AOAA or HMPSNE for 48 h and corresponding densitometry analysis (**A**–**D**). β-Actin was used as loading control. N = 3 independent experiments ± SEM.

We also assessed whether AOAA or HMPSNE affect the expression of these enzymes after incubation for 24 or 48 h. No statistically significant effects were detected (Figure 1 and Figure 2).

**Figure 3:**
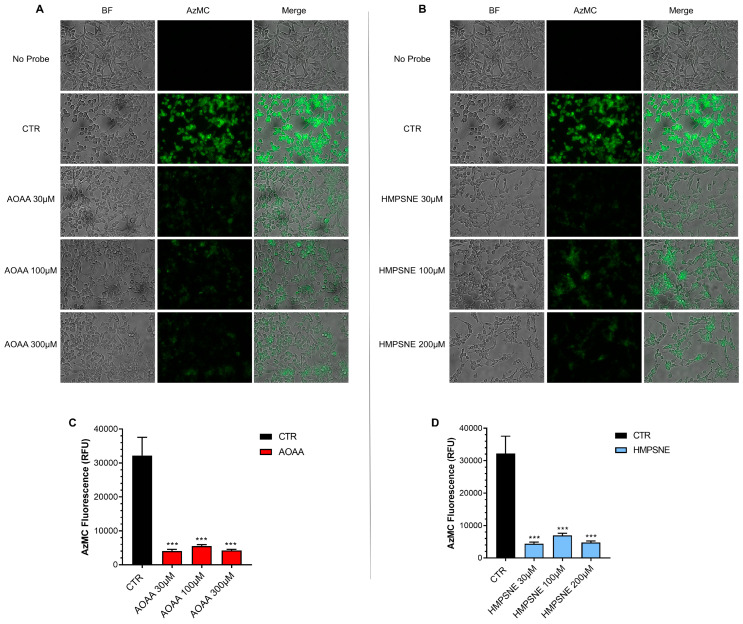
H_2_S synthesis in EO771 cells; effect of CBS/CSE or 3-MST inhibition. The H_2_S-synthesizing capacity of the cells was analyzed by live cell imaging. Incubation with the CBS/CSE inhibitor AOAA (**A**,**C**) or the 3-MST inhibitor HMPSNE (**B**,**D**) (24 h treatment of the cells) markedly reduced the H_2_S signal. N = 3 independent experiments ± SEM; *** *p* < 0.001 shows a significant inhibitory effect of AOAA or HMPSNE on the cellular H_2_S signal.

Using live cell imaging (with the utilization of AzMC, a fluorescent H_2_S dye) significant H_2_S generation was detectable in EO771 cells (Figure 3).

When treated with the CBS/CSE inhibitor AOAA (Figure 3A,C), or the 3-MST inhibitor HMPSNE (Figure 3B,D), H_2_S generation was markedly decreased. These data indicate that cellular H_2_S generation is dependent on both the CBS/CSE and the 3-MST pathways.

**Figure 4:**
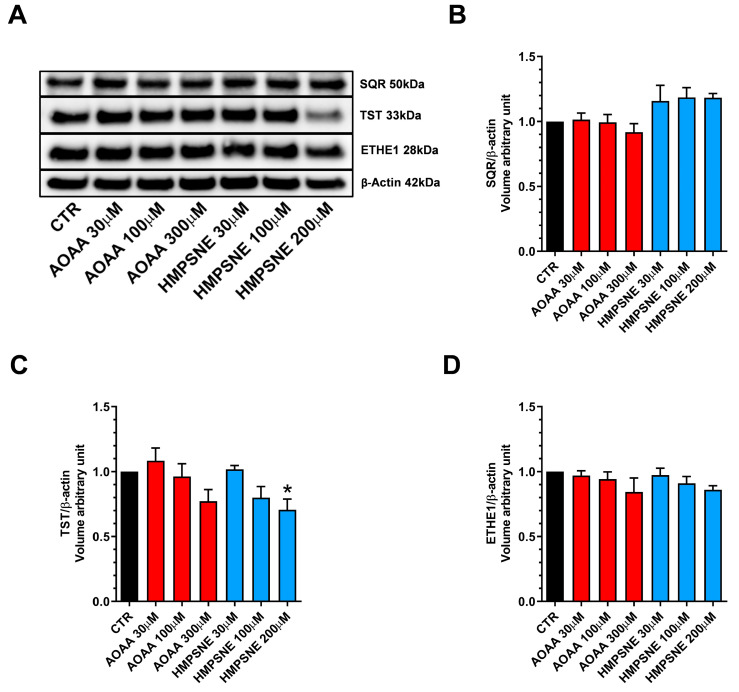
H_2_S-catabolizing enzyme expression in EO771 cells; effect of CBS/CSE or 3-MST inhibition. SQR, TST and ETHE-1 expression was analyzed by Western blotting (**A**) and densitometry analysis (**B**–**D**); in the absence or presence of 48 h of AOAA or HMPSNE treatment. N = 4 independent experiments ± SEM. * *p* < 0.05 indicates significant inhibition of TST expression by HMPSNE.

Expression of the known H_2_S-metabolizing enzymes, SQR, TST (rhodanese) and ETHE-1 was also detected in these cells (Figure 4).

Treatment of the cells with AOAA tended to slightly reduce the expression levels of all 3 enzymes (Figure 4A–D), while treatment of the cells with HMPSNE (200 µM) significantly reduced TST expression (Figure 4A,C). It is likely that the protein levels of H_2_S degrading enzymes are regulated by the ambient H_2_S levels: when the H_2_S levels are pharmacologically suppressed, the H_2_S degrading enzymes may, in turn, may become downregulated as a response.

### 3.2. Role of CBS and 3-MST in the Regulation of Cellular Bioenergetics in EO771 Cells

**Figure 5:**
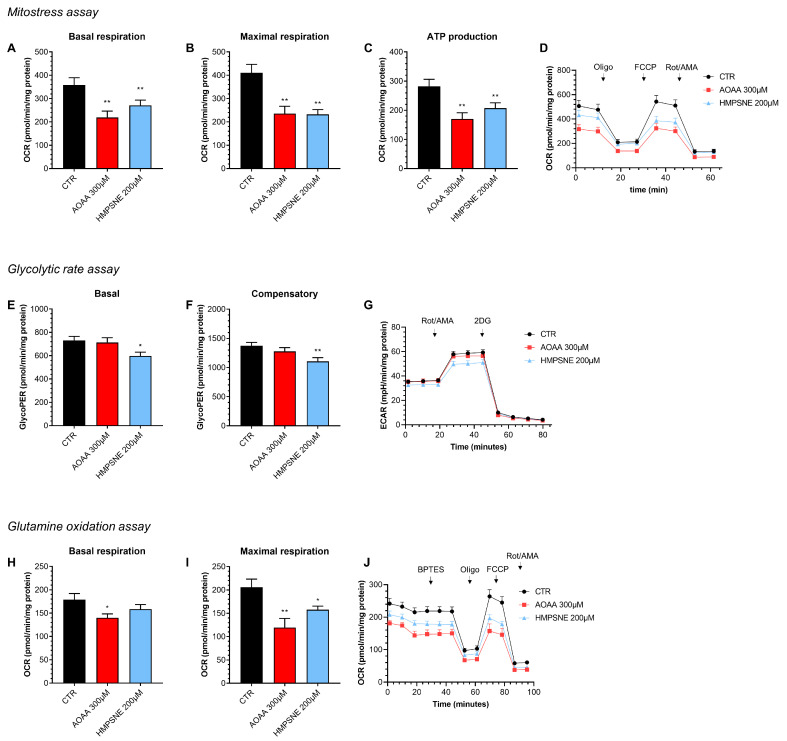
Effect of AOAA (300 µM) or HMPSNE (200 µM) on cellular bioenergetics in EO771 cells. The CBS/CSE inhibitor AOAA or the 3-MST inhibitor HMPSNE reduced mitochondrial electron transport and aerobic ATP generation when cells utilized glucose as their main substrate (**A**–**D**). HMPSNE also reduced glycolytic activity (**E**–**G**). Both inhibitors (AOAA or HMPSNE) also reduced mitochondrial function when cells were utilizing glutamine (rather than glucose) as their substrate (via glutaminolysis) (**H**–**J**). N = 3 independent experiments ± SEM; * *p* < 0.05 or ** *p* < 0.01 shows a significant inhibitory effect of AOAA or HMPSNE.

Using Extracellular Flux Analysis, we have next tested the role of endogenous H_2_S generation on the maintenance of cellular bioenergetics in EO771 cells, an effect that has previously been demonstrated in various other cancer cell types [12]. The results show that both the inhibition of CBS/CSE and of 3-MST suppresses basal oxidative phosphorylation/mitochondrial ATP generation as well as—in the case of 3-MST but not CBS/CSE—anaerobic ATP generation (glycolysis), when the cells are using glucose as their primary bioenergetic source (Figure 5).

When we use glutamine as a bioenergetic source (instead of glucose), once again, both the 3-MST inhibitor and the CBS/CSE inhibitor suppress mitochondrial oxygen consumption and ATP generation (Figure 5). Based on these data, endogenously generated H_2_S, largely independently of its source (CBS/CSE vs. 3-MST) and largely independent of the substrate used by the cell (e.g., glucose vs. glutamine) plays a role in the maintenance of basal aerobic (i.e., mitochondria-dependent) bioenergetic function. With respect to basal cellular glycolysis, 3-MST, but not CBS/CSE appears to play a significant role.

### 3.3. Role of CBS and 3-MST in the Regulation of Proliferation and Migration in EO771 Cells

**Figure 6:**
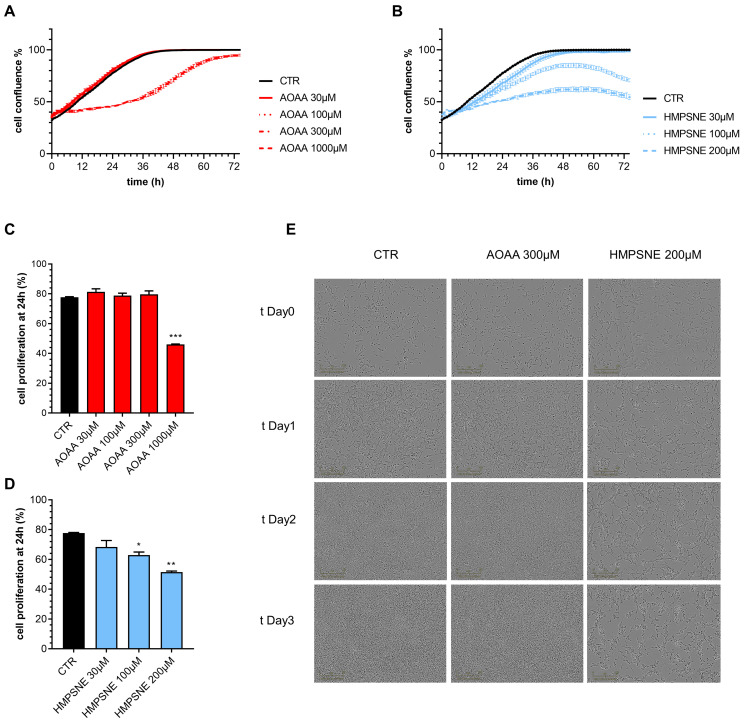
Effect of inhibition of H_2_S biosynthesis on EO771 cell proliferation. (**A**,**B**): Proliferation curves of E0771 cells treated with AOAA (**A**) or HMPSNE (**B**). Cells were monitored over 72 h. Quantification of cell confluence reached after 24 h of E0771 cells treated with AOAA (**C**) or HMSNE (**D**). Representative images of 3 days treatment obtained with IncuCyte ZOOM €. N = 3 independent experiments ± SEM; * *p* < 0.05, ** *p* < 0.01 and *** *p* < 0.001 show a significant inhibitory effect of AOAA or HMPSNE.

We have next assessed whether pharmacological inhibition of CBS or 3-MST affects proliferation and migration of EO771 cells. Inhibition of the CBS/CSE axis reduced cell proliferation, but only at its highest concentration (1000 µM) tested; the observed effect was transient, and at later time points (>24 h) cells appeared to regain a faster rate of proliferation; by 72 h, cell confluence was comparable in all groups with or without AOAA (Figure 6A,C,E).

In contrast, HMPSNE produced a concentration-dependent and sustained inhibition of cell proliferation throughout the 72 h observation period (Figure 6B,D,E). The effect of the 3-MST inhibitor remained pronounced and statistically significant at the conclusion of the experiments at 72 h (Figure 6B,D,E).

**Figure 7:**
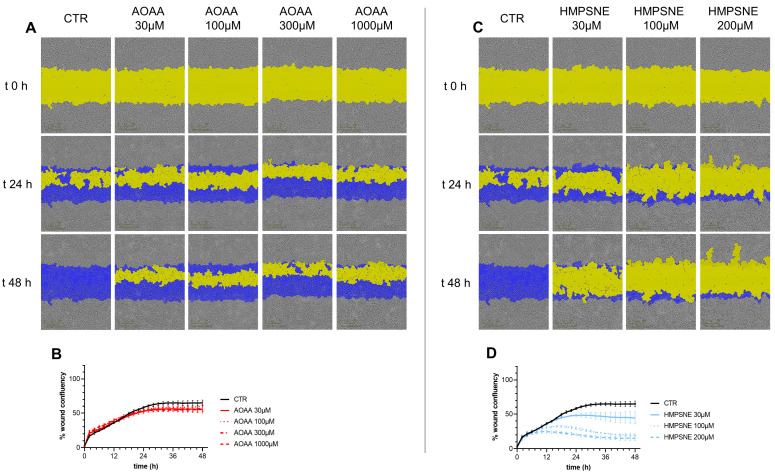
Effect of inhibition of H_2_S biosynthesis on EO771 cell migration. Cells were treated with various concentrations of AOAA (**A**,**B**) or HMPSNE (**C**,**D**). Images (**A**,**C**) show closure of the wound for up to 48 h post wounding. (Scratch wound mask in yellow, initial scratch wound mask in blue). Panels B and D show percent wound closure of EO771 cells treated with increasing concentrations of AOAA (**B**) or HMPSNE (**D**) over time; mean ± SEM values, N = 3. The effect of HMPSNE (all three concentrations tested) was statistically significant, *p* < 0.05.

Cell migration was not significantly affected by treatment with AOAA (Figure 7A,B). In contrast, HMPSNE exerted a concentration-dependent inhibitory effect (Figure 7C,D).

### 3.4. Role of CBS and 3-MST in the Regulation of Oxidative Stress Response in EO771 Cells

**Figure 8:**
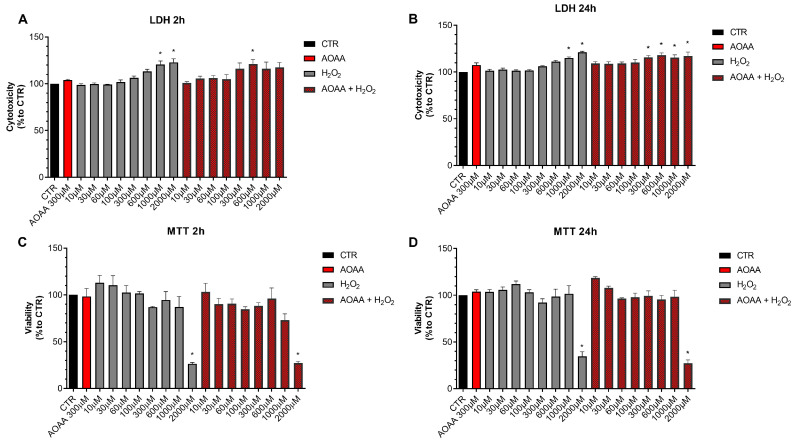
Effect of AOAA on the response to oxidative stress in EO771 cells. The CBS/CSE inhibitor (300 µM) did not affect oxidative stress induced cell death/loss of cell viability measured by LDH release (**A**,**B**) or MTT conversion (**C**,**D**) in the presence of H_2_O_2_ challenge, as assessed at 2 or 24 h. N = 3 independent experiments ± SEM. * *p* < 0.01 shows significant differences compared to untreated control values.

Next, we determined if pharmacological inhibition of H_2_S biosynthesis affects the response to oxidative stress in EO771 cells. Using the LDH and MTT assays, the effect of the rapid acting cytotoxic oxidant hydrogen peroxide (H_2_O_2_) was tested. This oxidant, as expected, decreased cell viability in a concentration-dependent fashion. When cells were pretreated with AOAA, the response to the oxidant was unaffected (Figure 8).

**Figure 9:**
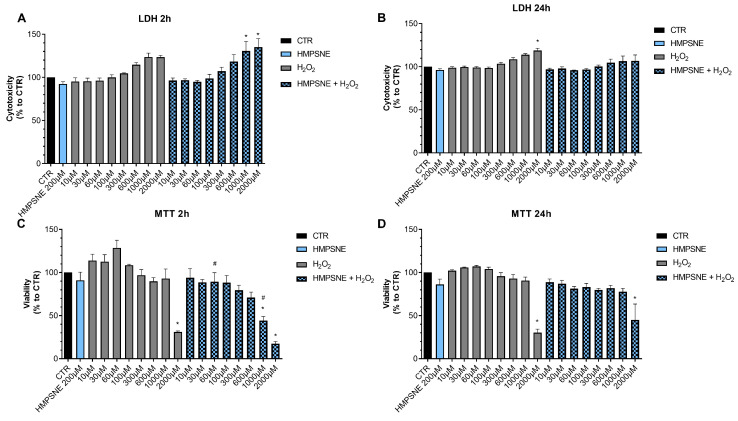
Effect of HMPSNE on the response to oxidative stress in EO771 cells. The 3-MST inhibitor (300 µM) did have a marked overall effect on oxidative stress induced cell death/loss of cell viability measured by LDH release (**A**,**B**) or MTT conversion (**C**,**D**), as assessed at 2 or 24 h. Nevertheless, at 2 h, in the presence of at 60 and 1000 µM H_2_O_2_, HMPSNE tended to exacerbate the degree of cell dysfunction, assessed by the suppression of MTT converting-ability (# *p* < 0.05 represents significant difference in MTT or LDH at the same concentration of H_2_O_2_, in the presence vs. absence of 300 µM HMPSNE). N = 3 independent experiments ± SEM. * *p* < 0.05 shows significant effects of H_2_O_2_ compared to untreated control values.

In contrast to the lack of effect of AOAA, the 3-MST inhibitor HMPSNE tended to have a slight potentiating effect on the response to oxidative stress, but this effect was only statistically significant at 2 h and not 24 h, and only statistically significant at certain concentrations of the oxidant (Figure 9). For instance, in the presence of the 3-MST inhibitor, the ability of H_2_O_2_ (1000 and 2000 µM) to increase LDH release was more significant than in the absence of the inhibitor (Figure 9A). Moreover, the ability of 60 or 1000 µM H_2_O_2_ to suppress cellular MTT conversion to formazan was statistically more pronounced (Figure 9C).

These data indicate that H_2_S may play a slight role as a cytoprotective agent in breast cancer cells, but this effect is fairly minor, and is only detectable for 3-MST-derived H_2_S or polysulfides, but not for CBS/CSE-derived H_2_S. AOAA or HMPSNE, on its own, at the concentrations used, did not have any marked effects of cell viability in non-oxidatively stressed cells (Figure 8 and Figure 9).

## 4. Discussion

In 2013, it was demonstrated that colon cancer cells show increased expression of CBS, and use its product, H_2_S, to support their cellular bioenergetics, proliferation, growth, and angiogenesis [13]. Follow-up studies in colon cancer, as well as in a variety of other cancers including ovarian cancer, glioblastoma, and lung cancer, have confirmed and extended these observations [14,15,16,17,18,19,20]. The enzymatic source of H_2_S was found to be CBS in many cancer types; but in some models, CSE and/or 3-MST were found to contribute as well [3,7,15,19,21,22,23,24,25,26].

With respect to breast cancer, the first report was published by Sen and colleagues in 2015, demonstrating that CBS is upregulated in human breast cancer cell lines, H_2_S is produced in excess, and it serves as a tumor cell-supporting mediator [27]. Among others, this report demonstrated that H_2_S serves as a factor that protects breast cancer cells from macrophage-mediated cytotoxicity and elimination [27,28]. Importantly, in murine xenograft models, breast cancer cell growth was markedly slower in CBS-silenced cells than the growth of wild-type cells [27]. Subsequent studies, specifically focusing on triple-negative human breast cancer cells in vitro, demonstrated that exogenously administered H_2_S exerts a bell-shaped effect, with lower concentrations of the mediator promoting proliferation, migration and colony formation, while higher concentrations induce the opposite effects [29]. This bell-shaped concentration-response is characteristic of various gaseous mediators including H_2_S and also supports the development of multiple therapeutic concepts either based on inhibition of endogenous H_2_S generation or delivery of exogenous H_2_S to produce anticancer cell toxicity [1,2].

Subsequent studies on H_2_S in breast cancer cells focused on the identification of the endogenous enzymatic sources of this gasotransmitter, as well as on the effects it exerts. Wang and colleagues, using MDA-MB-231 human breast cancer cells (a triple-negative line) identified CSE as a key source of H_2_S and demonstrated that the metastatic ability of these cells is, at least in part, dependent on the CSE/H_2_S axis and also involves VEGF signaling and PI3K activation [30]. Later on, the work of Nagy and colleagues demonstrated that both CBS and CSE expression are important to support breast cancer cell proliferation and survival. This work primarily focused on basal-like breast cancer, an aggressive cancer subtype. These studies demonstrated that CBS silencing (shCBS) makes these cells less invasive, reduce their proliferation rate, renders them more vulnerable to oxidative stress and cystine deprivation, sensitizes them to ferroptosis, and renders them less responsive to HIF1-α activation under hypoxia [31].

The current work, utilizing a murine breast cancer cell line, focused on the expression of all major H_2_S-generating and H_2_S-metabolizing enzymes and tested the effect of pharmacological inhibition of CBS/CSE vs. 3-MST on a variety of functional parameters. While EO771 cells expressed all three major H_2_S-generating enzymes CBS, CSE, and 3-MST, and produced significant levels of H_2_S due to a combination of these enzymes, the pharmacological experiments (using the combined CBS/CSE inhibitor AOAA or the 3-MST inhibitor HMPSNE) revealed a more pronounced role of 3-MST in these cells than CBS or CSE. Regarding the bioenergetic aspects, both AOAA and HMPSNE tended to suppress various bioenergetic parameters related to oxygen-dependent ATP generation (i.e., mitochondrial function), while only the 3-MST inhibitor was found to suppress the glycolytic activity of these cells. When comparing the functional responses to these pharmacological agents, the CBS/CSE inhibitor only exerted a transient and relatively slight inhibitory effect on cell proliferation (and this effect was only noted at a high concentration of this agent, at which concentration effects on additional enzymatic targets may also possible [10]), and had no inhibitory effect on cell migration, nor did it affect the cell’s responsiveness to exogenously administered hydrogen peroxide. In contrast, the 3-MST inhibitor exerted a concentration-dependent, marked inhibitory effect both on cell migration and cell proliferation, and it also tended to exacerbate the response to the oxidant, although this effect was only noted at certain concentrations of H_2_O_2_ and only in the short-term, but not the longer-term experiment. Taken together, it appears that in EO771 cells, H_2_S generation from 3-MST plays a more prominent cancer-cell-supporting role, and the functional role of CBS and/or CSE is relatively minor. In this respect, EO771 cells appear to resemble the CT26 murine colon cancer cell line, in which also 3-MST (rather than CBS or CSE) appears to play the primary tumor-cell-supporting role [7]. Whether the more prominent role of 3-MST in murine cells (as opposed to CBS and/or CSE in human cells) represents a more general trend remains to be investigated in the future.

The current study, thus, confirms and extends the growing body of information regarding the functional importance of endogenously generated H_2_S in breast cancer cells. Clearly, it has several limitations. First of all, it is a strictly in vitro study and does not incorporate tumor-bearing mouse models. (Notably, however, these cells are on a Bl6 background and will be amenable for such experiments in the future. Such experiments may be used in the future to compare the relative importance of tumor-cell-derived vs. host-derived H_2_S in the modulation of breast cancer cell growth.) Second, it primarily focuses on basal fundamental functional parameters (e.g., bioenergetics and cell proliferation and migration) and does not utilize more complex models (e.g., cancer cell death induced by anticancer agents or immune cell co-cultures). Third, it only utilizes one selected cell line (it is possible that the H_2_S-producing enzyme expression profile of other murine breast cancer cell lines is different from those characterized here). Fourth, it does not investigate the functional role of the H_2_S-degradation pathways, only focuses on the H_2_S production aspect. These enzymes SQR, TST and ETHE-1, indeed, play an important role in the modulation of intracellular H_2_S levels, by significantly affecting the rate of H_2_S degradation [1,2]. The reason for less focus on these enzymes in the current project is that we believe that inhibition of these enzymes is more translationally relevant than modulation of the degradation pathways; another reason is that the availability of cell-permeable small molecules to modulate the degradation pathways is rather limited. Fifth, the pharmacological agents used in the current study, AOAA and HMPSNE, have their own limitations. Regarding AOAA, the mechanism of its action has recently been overviewed, as well as its selectivity profile [10]. This compound, although commonly referred in the literature as a ‘CBS inhibitor’, also inhibits CSE [5] as well as many PLP-dependent enzymes [10]. Moreover, it is commonly referred as an ‘irreversible inhibitor’, although recent data reveal that its inhibitory effect on CBS can be also reversible under certain conditions [32]. With respect to its effect on H_2_S generation, we provide in the current report direct evidence that the compound, indeed, reduces cellular H_2_S levels (as shown by its effect on AzMC-aided H_2_S detection in live cells), and we also demonstrate that it does not affect the expression of the principal H_2_S-generating or H_2_S-metabolizing enzymes (as quantified by Western blotting). While it may have additional enzymatic targets beyond the H_2_S pathway, overall, its functional effects in the current system were minor. Thus, we feel that we can conclude with confidence that CBS and CSE in the current cell line only play a minor functional role. With respect to the 3-MST inhibitor used (HMPSNE), the availability of pharmacological 3-MST inhibitors is rather limited, and from the limited choices (which, in previous years, utilized non-specific compounds such as L-aspartic acid) this compound is vastly superior and is used in the literature fairly commonly, and without evidence of significant off-target or non-specific effects [7,11,33,34,35,36,37,38,39,40,41,42]. Using this compound (at the concentration range that is comparable to those used in prior studies), a significant functional role of the 3-MST/H_2_S pathway could be demonstrated in the current cellular model.

Nevertheless, the utilization of pharmacological agents (in general, and also in particular in the current set of experiment) may be complicated by non-specific (“off-target”) effects. Therefore, further studies remain to be conducted in the future to further validate the findings. Such studies may include silencing/knockout approaches (e.g., siRNA, shRNA or CRISPR) for CBS or 3-MST, with the caveat that these approaches may induce significant cytotoxicity due to the long-term absence of enzymes that may be crucial for cancer cell survival. Indeed, long-term treatment with 3-MST inhibitors have been shown to promote cancer cell apoptosis [42].

The current findings are solely in vitro. Future, in vivo studies remain to be conducted to test the effect of CBS or 3-MST inhibition in mouse models bearing breast cancer cell lines. Based on prior in vivo studies using other forms of cancer (e.g., colon cancer, ovarian cancer, or other forms of breast cancer) [4,31,38] we anticipate that inhibition of CBS or 3-MST will suppress cancer cell proliferation.

## 5. Conclusions

In summary, endogenous H_2_S, primarily produced by 3-MST (and to a minor degree by CBS/CSE), significantly contributes to the maintenance of bioenergetics, proliferation and migration in the murine breast cancer cell line EO771 and may also exert a minor role as an endogenous cytoprotectant. Given the physiological role of the same enzyme in maintaining and supporting various fundamental metabolic functions also in normal healthy tissues [40], further studies are required to determine if inhibition of 3-MST, on its own, or in combination with various anticancer therapeutics, may be useful to limit the growth and proliferation of breast cancer cells in vivo.

## Data Availability

Data pertinent to the current study will be deposited in a data repository complying with the FAIR Data Principles. Data will also be provided, upon request, to qualified investigators by the author of correspondence.
